# Athlete's heart and other entities to consider around

**DOI:** 10.1002/clc.23464

**Published:** 2020-09-24

**Authors:** María Martín, Antonio Adeba

**Affiliations:** ^1^ Cardiology Department Central University Hospital of Asturias Oviedo Spain; ^2^ Instituto de Investigación Sanitaria del Principado de Asturias (ISPA) Oviedo Spain

Dear Editor,

We have read with great interest the manuscript recently published in your journal by Malek and Bucciarelli‐Ducci entitled “Myocardial fibrosis in athletes—Current perspective.”^1^ We would like to make some considerations about it and to highlight another several aspects that we think could be also of interest. First of all, left ventricle noncompaction cardiomyopathy (LVNC) is a progressive cardiomyopathy associated with an increased risk of embolic events, sudden death, and heart failure related to left ventricle dysfunction. Distinguishing between LVNC and hypertrabeculation secondary to exercise is fundamental in athletes and has been a matter of controversy and publications^2^ Hypertrabeculation in athletes can be therefore a normal variant and probably it should be considered and included as another expression of the athlete's heart; however, differential diagnosis with LVNC can be sometimes extremely difficult and some aspects referred by Malek and Bucciarelli‐Ducci like family background, genetic testing, and left ventricle dysfunction with thinning myocardial areas can help in the diagnosis. In this sense, Gati et al investigated the prevalence of increased left ventricular trabeculation in highly trained athletes.^3^ They found that a high proportion of young athletes had conventional criteria for LVNC and that it was even most common in African and Afro‐Caribbean athletes so, in these cases, diagnosis of LVNC should be cautious and other criteria like the above mentioned and others such as left ventricular systolic function, marked repolarization changes in Electrocardiogram and, of course, fibrosis in Magnetic Resonance Imaging should be always take into account. Related to this, another final question arises about which should be the stratification criteria of sudden death for LVNC. Further studies and international registers are needed in order to elaborate an appropriate score of sudden death risk in these patients.

Otherwise, another entity we would like to mention and that we think should be included separately by Malek and Bucciarelli‐Ducci is *isolated arrythmogenic left ventricle dysplasia*. Predominant left ventricular involvement with fibro‐fatty infiltration and replacement and without affecting right ventricle is rare, and there is a lack of specific criteria for it which can make its diagnosis sometimes difficult. Magnetic Resonance Imaging findings include fibrosis with left ventricle late gadolinium enhancement in a subepicardial/midwall distribution and then differential diagnosis include previous myocarditis A high level of suspicion of this under‐recognized cardiomyopathy should be present and again and, due to the high risk of sudden death, other factors such as familial disease, systolic dysfunction, and Electrocardiogram changes must be always considered.^4^

## CONFLICT OF INTEREST

The authors declare no potential conflict of interest.
